# Impacts of the removal of shrubs on the physiological and biochemical characteristics of *Syntrichia caninervis* Mitt: in a temperate desert

**DOI:** 10.1038/srep45268

**Published:** 2017-04-04

**Authors:** Ben-feng Yin, Yuan-ming Zhang, An-ru Lou

**Affiliations:** 1 State Key Laboratory of Earth Surface Processes and Resource Ecology, College of Life Sciences of Beijing Normal University, Beijing 100875, China; 2 Xinjiang Institute of Ecology and Geography, Key Laboratory of Biogeography and Bioresources in Arid Land, Chinese Academy of Sciences, Urumqi 830011, China

## Abstract

Moss crusts play important roles in biological soil crusts biomass and soil surface stabilization. However, because of increasingly intensive human activities, especially grazing, the growth and survival of shrubs are seriously threatened. This study aimed to test whether the presence of shrubs affects the physiological state of the bryophyte *Syntrichia caninervis* Mitt. in this desert ecosystem. We simulated animal-grazed shrubs at three levels in the Gurbantunggut Desert and compared these simulations to exposed areas, measuring the indicators of growth and stress tolerance exhibited by bryophytes. The results showed that the removal of shrubs significantly decreased chlorophyll fluorescence activity and soluble protein content in *S. caninervis*, especially under the total shrub removal treatment. The ratio between the total removal of shrubs and other treatments in antioxidative enzymes and in osmotic adjustment substances of *S. caninervis* exhibited two types of responses. With the exception of malonyldialdehyde (MDA) and superoxide dismutase (SOD), the variables examined fitted as downward parabolic then upward parabolic temporal dynamics. The removal of shrubs is harmful to the survival of *S.caninervis*. In resource-constrained conditions, SOD is an important antioxidant enzyme that of peroxidase (POD), catalase (CAT) and osmotic adjustment substances, for *S. caninervis* survival.

In the arid and semi-arid desert regions of the world, biological soil crusts (BSCs) are often widely developed. The cover of BSCs can reach 70% in some desert ecosystems^1^,^2^. Moss-based crusts serve as the best-developed stage in the ecological succession of BSCs. They also play an important role in micro-ecosystems and constitute the majority of crust biomass^3^,^4^. BSCs play an important role in stabilizing the soil surface, changing the micro-environment and promoting vegetation succession^5^,^6^,^7^,^8^. However, BSCs are under threat in some areas because of intensive human activities, including overgrazing, oil and gas exploration, and land use change. These human activities have severely damaged the original desert landscape, leading to greatly reduced ecological functions^9^,^10^. The devastation of biocrusts can accelerate the process of desertification. However, the natural recovery is extremely difficult, requiring several years or even decades^9^,^11^.

To adapt to the extreme environment, desert bryophytes have developed strong adaptability in resistance to drought^12^,^13^,^14^, extreme temperatures^15^,^16^,^17^, and UV-B radiation^18^,^19^,^20^. A cushion cluster of a population can enhance the water retention capacity of the capillary system by reducing the air speeds above the plant surface and decreasing the amount of water evaporation^21^. Previous work suggests that leaves of desert bryophytes can be strongly rolled and tightly bound to the stem, greatly decreasing moisture evaporation and protecting the plants from strong radiation^22^,^23^. Additionally, in their leaves, bryophytes have evolved intensive and transparent papillae in their hair points and coast, which not only reflect strong light but also favor water collection and transportation^24^. In acutely cold environments, the cells of bryophytes can rapidly dehydrate and successfully prevent damage as result of ice accumulation in cells^25^.

In contrast to other vascular plants, bryophytes are poikilohydric organisms, in which the water content of cells is in equilibrium with the environment^26^,^27^. Many studies have demonstrated that the dormant bryophyte can completely recover its chlorophyll fluorescence activity in one or two hours and repair its protein fractions and cellular structure within 24 hours^12^,^28^,^29^. The free proline, soluble sugar and other osmotic adjustment substances of cells are accumulated in dehydration. These substances can be used to stabilize the cellular osmotic pressure, maintain the protein structure and normalize intracellular macromolecules in membrane structures^30^,^31^,^32^,^33^. In addition, the redundant oxygen and peroxides can be eliminated by synthesizing superoxide dismutase (SOD), peroxidase (POD), catalase (CAT) and other antioxidant enzymes^34^,^35^,^36^,^37^. These osmotic adjustment substances and antioxidants can also be synthesized by plants when they are subjected to salt stress, heavy metal pollution, UV-B radiation and other stress environments^38^,^39^,^40^,^41^. Studies have also shown that the responses to UV-B radiation involve the increase of the relative proportions of photo-protective compounds in plants, such as β-carotene, zeaxanthin, and violaxanthin^18^,^42^. But the responses of different bryophyte species to UV-B radiation increases are different^43^. In general, UV-B radiation will increase DNA damage and lead shoots to becoming stunted, and reduce annual growth and aboveground biomass of plants^44^,^45^.

Patchily distributed biological soil crusts and shrubs are the main vegetation cover types in many deserts. This patchy distribution results in some change in various survival environments of bryophytes, and whether these different microhabitats result in physiological or genetic differences between bryophytes remains controversial. Two lines of evidence have addressed this question. First, some research suggest that short distances between extreme microhabitats do not result in ecotypes, although there are considerable morphological differences^46^. Second, some studies have found that there are substantial differences between different habitats in terms of sex ratios^12^,^35^, reproduction modes^47^, and physiological state^36^,^48^ among variables. Previous studies found that male bryophytes rarely were living in exposed areas, and stress tolerance of bryophytes may differ among different habitats. Plants distributed in exposed areas possess better adaptability to the harsh environment than those under shrubs^49^,^50^. Therefore, in the present study, we attempted to eliminate the differences between habitat types in our experiments.

In contrast to other deserts, the Gurbantunggut Desert receives abundant snowfall during the winter – approximately 20 cm depth. The snow cover as an insulator exerts wetting and warming effects to mosses^51^, and snow thaw in the spring provides appropriate conditions for the growth of bryophytes and for the germination and growth of annual plants^52^,^53^. In recent years, however, with the introduction of mining and livestock, rodents such as gerbils are beginning to breed rapidly, resulting in the severe destruction of shrubs and soil surfaces. In dry years, there are fewer ephemeral, ephemeroid and annual plants. Tree species, such as *Haloxylon persicum* and *Haloxylon ammodendron*, as well as certain dwarf shrubs, specifically *Ephedra distachya*, are the main food source and hoarding objects. Thus, the objectives of this study were to explore whether substantial gnawing or destruction will cause serious injury to bryophytes that live under shrubs. Furthermore, we aimed to identify whether stable snow cover could protect the plants from acute cold temperatures during the winter. Given the loss of shrubs providing protection, we hypothesized that the chlorophyll fluorescence and soluble protein content would be reduced in conjunction with the removal of shrubs. Correspondingly, stress-related indices, such as the osmotic adjustment substance content and antioxidative enzyme activity, were expected to increase with time and the removal of shrubs.

## Results

To better reveal the effects of the removal of shrubs on *Syntrichia caninervis*, we measured the indicators during the snow cover periods, freeze-thaw periods, and dry seasons in April and August for the four traditional types of desert climates - in other words, at 2 months, 4 months, 5 months and 9 months after the removal of shrubs.

### Chlorophyll fluorescence

**Figure 1:**
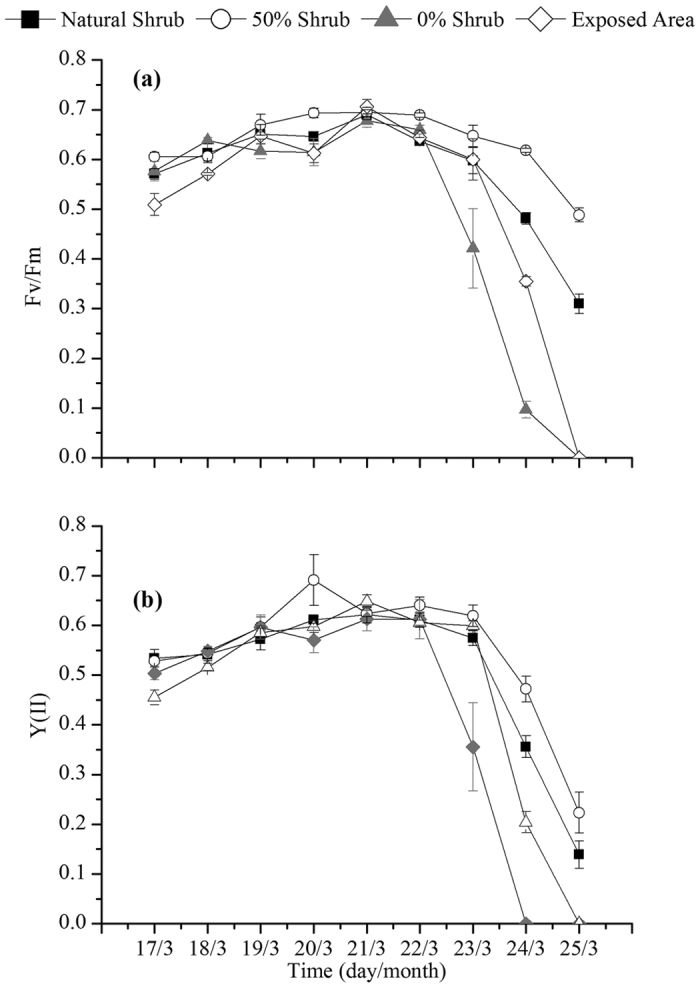
The variations in the maximal photochemical efficiency of PS_II_ (Fv/Fm) and the actual PS_II_ efficiency (Y(II)) in *Syntrichia caninervis* shoots during the freeze-thaw periods in different treatments.

**Figure 2:**
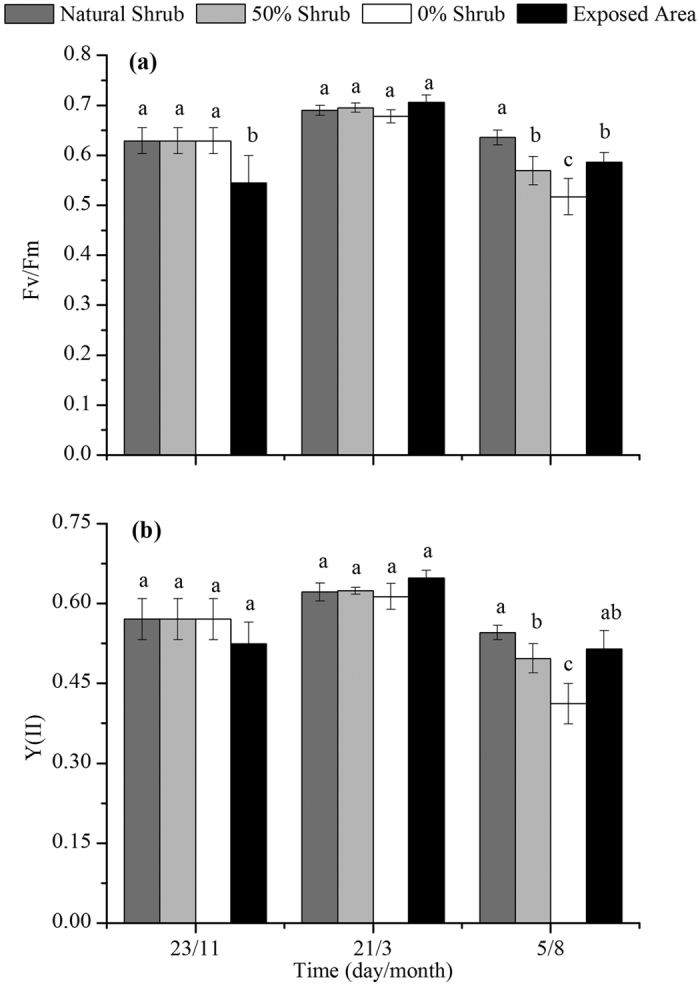
Changes in chlorophyll fluorescence in *Syntrichia caninervis* shoots in different treatments. The bars represent the means of five replications ± the standard error. Different letters within each treatment indicate a significant difference (*P* < 0.05).

The removal of shrubs did not exert significant influences on the chlorophyll fluorescence of *Syntrichia caninervis* compared with the control during the winter. However, there were obvious differences in the duration of chlorophyll fluorescence activity during the freeze-thaw periods. The duration of the chlorophyll fluorescence activity of *Syntrichia caninervis* with the 0% shrubs was shorter than that for the 50% shrubs, for the natural shrubs and even in exposed areas (Figs 1 and 2).

The results of the repeated-measures ANOVA showed that chlorophyll fluorescence activity, which included the maximal photochemical efficiency of PS_II_ (Fv/Fm) and the actual PS_II_ efficiency (Y(II)), was affected by the period and interaction between the period and habitat. The habitat (removal of shrub) significantly affected the Fv/Fm while exerting no impacts on Y(II). The Fv/Fm and Y(II) values of *Syntrichia caninervis* for the 0% and 50% shrub groups were significantly lower than that in the natural shrub group after 9 months of treatment (Fig. 2).

### Osmotic adjustment substances

**Figure 3:**
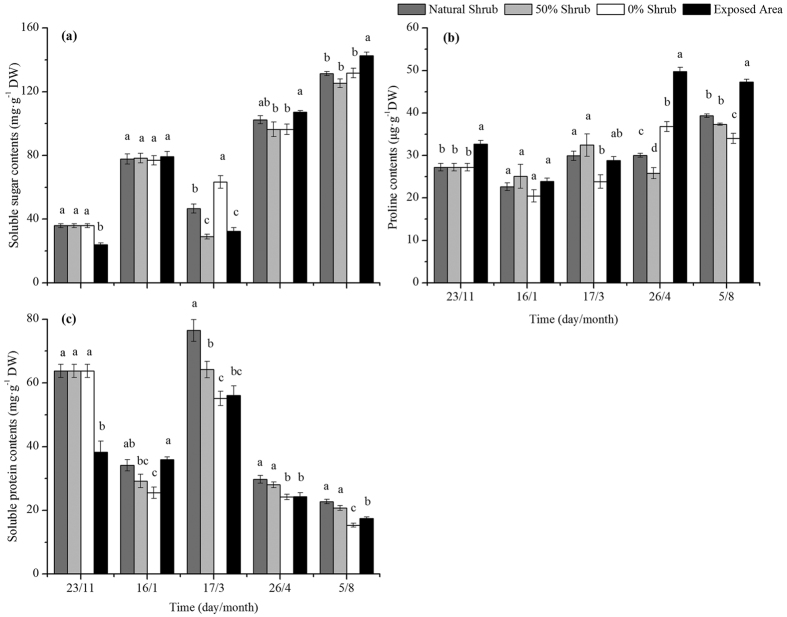
Changes in the content of proline, soluble sugar and soluble protein in *Syntrichia caninervis* shoots in different treatments. The bars represent the means of five replications ± the standard error. Different letters within each treatment indicate a significant difference (*P* < 0.05).

The periods, habitat and their interaction significantly affected the free proline, soluble sugar and soluble protein content (Table 1). The soluble sugar content for the removal treatment plots was significantly higher than that of the natural shrub plots and exposed areas during the freeze-thaw periods. However, in dry periods, the soluble sugar content in the 0% shrub plots was not significant compared with the natural shrub plots, nor were there significant effects in the exposed areas. Unlike the soluble sugar content, the proline content in the 0% shrub plots continued to decrease after snowmelt. In addition, the proline content was lower than that in the exposed areas. And the proline content in the 0% shrub plots was significantly lower than in the 50% and natural shrub plots after 9 months of treatment. However, the soluble protein content in the natural shrub plots was significantly higher than in the 50% and 0% shrub plots, whereas the 0% shrub plots were also significantly lower than the exposed areas during the snow cover and dry periods (Fig. 3c).

### Antioxidant enzyme

**Figure 4:**
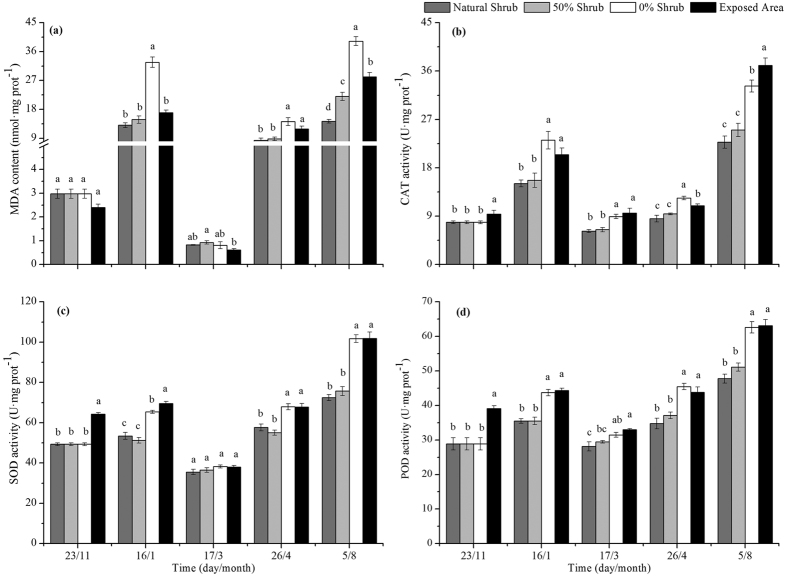
Changes in the content of malonyldialdehyde (MDA), the activity of peroxidase (POD), superoxide dismutase (SOD) and catalase (CAT) in *Syntrichia caninervis* shoots in different treatments. The bars represent the means of five replications the standard error. Different letters within each treatment indicate a significant difference (*P* < 0.05).

Compared with the 50% and natural shrub plots, the MDA content in the 0% shrub plot was significantly higher, except during the freeze-thaw periods (Fig. 4a), whereas the MDA content in the 50% shrub plots increased compared with the natural shrub plots. With the corresponding MDA, the activity of the antioxidant enzymes (e.g., POD, SOD, CAT) tended to be significantly higher than that in 50% and natural shrub plots. Although there was no significant difference between the 0% shrub plots and exposed areas in most measurements, the CAT activity was significantly lower in the 0% shrub plots after 9 months of treatment.

### Regression between physiological state and removal time

Data from winter were not incorporated into the analysis because the snow cover may result in deviations in the parameters associated with the activity of *Syntrichia caninervis*, including the Fv/Fm, Y(II) and soluble protein content. In response to removal time, these deviations were well documented by a linear relationship (Table 2). In addition, with the increasing time of shrub removal the activity of *Syntrichia caninervis* was decreased. Most of the physiological state associated with stress resistance, involving osmotic adjustment substances and antioxidant enzymes in response to removal time, were well documented by second-order polynomial regressions (Table 2). Except for the MDA and SOD activity, the fitted downward parabola and upward parabola elucidated temporal dynamics for other substances. These findings suggested that the contents of the osmotic adjustment substances and activity of the antioxidant enzymes exhibit an initial increasing and then decreasing pattern.

## Discussion

In the Gurbantunggut Desert, overgrazing, oil and gas exploration and the excessive breeding of gerbils have seriously disturbed the soil surface and decreased vegetation. Furthermore, these human activities have affected the growth of cryptogams living under shrubs. In addition, other factors, including sand burial, may have important effects on the physiological and biochemical characteristics of the vegetation.

### The effect of the removal of shrubs on chlorophyll fluorescence activity

The removal of shrubs significantly affected the Fv/Fm and Y(II) of *Syntrichia caninervis*, especially under the complete removal treatment. These results are partially consistent with the hypothesis that the removal of shrubs will adversely affect the short-term growth of bryophytes. The direct effect of shrub removal is the loss of vegetation protection for cryptogams. This protection includes shielding from sunlight, preventing trampling and sand burial. The presence of shrubs can form a protected area at the center of the shrub canopy, protecting the bryophytes from disturbances due to grazing to a limited extent^9^. Shrubs also have important fertility and moisture island effects, which result in a visible increase in fine particles, soil organic matter and nutrients, reduced water evaporation, and a favorable local microenvironment^54^,^55^. Moreover, the negative impacts on the growth of bryophytes from UV-B radiation are decreased due to the shelter provided by shrubs^20^.

Our results show that the Fv/Fm and Y(II) of *Syntrichia caninervis* consistently decreased with time after shrub removal. The Fv/Fm reflects the solar energy absorbance in the PS_II_ reaction center by plants for the maximum efficiency of the photochemical primary reaction. Fv/Fm represents the activity of the PS_II_ reaction center^56^,^57^, whereas Y(II) reflects the direct response of plants in actual photochemical efficiency when plants are in a stressful environment. Recent studies reported that the Fv/Fm, Y(II), ETR, and chlorophyll contents reduced after destruction of microhabitats, and the findings of the present study are consistent with those of previous studies^58^. According to the analysis of fitted curves, these deviations were well documented by a linear relationship in response to removal time, and with increasing the time after removal the activity of *Syntrichia caninervis* decreased. With the removal of shrubs, the “shade” and “fertile island” effects will disappear^48^. To the best of our knowledge, there is no evidence of fatal threat to these mosses when subjected to temperature, moisture or light intensity stress alone^58^,^59^. For example, temperature stress alone temporarily reduced Fv/Fm activity of bryophytes to acclimate to the environment^59^,^60^. However, such stress caused by removal of shrubs is usually accompanied by high light intensity and drought in the field, potentially making conditions lethal^61^. For a long term, of course, the activity may be restored with vegetation restoration^62^.

Light transmittance decreases with the increasing depth of snow, and snowmelt in spring provides sufficient water for plant growth. Freeze-thaw periods in spring are the main growing season for biocrusts in the Gurbantunggut Desert^63^. This may be one important reason for the similar maximum chlorophyll fluorescence activities between treatments. Stable snow cover during the winter in Gurbantunggut Desert may play a similar role as shrubs in protecting *Syntrichia caninervis* from intense radiation. Although the removal of shrubs had no significant effects on the chlorophyll fluorescence activity during the winter, the duration of activity is greatly reduced and even lower than that in exposed areas (Fig. 1). This is likely a result from the rapid water evaporation as the shrubs were removed. It is worth mentioning that the duration of chlorophyll fluorescence activity of *Syntrichia caninervis* under 50% shrubs was higher than that in natural shrubs, during the snowmelt periods. The ratio of MDA content between 0% shrubs and natural or 50% shrubs also decreased slightly in this period. This indicated that partial removal of shrubs may contribute to the bryophyte *Syntrichia caninervis* growth during the snowmelt periods. Marschall and Proctor^64^ studies suggest that the bryophyte is not a natural shade plant, but strong sunlight will cause harm to plants. There is, to our knowledge, no evidence of bryophyte growth in a favorable direction caused by removal of shrubs during other seasons.

It has been well established that increased UV-B radiation has harmful effects on the rate of photosynthesis of many plants^61^. Laboratory experiments have indicated that the effects of UV-B radiation on plant photosynthetic systems are primarily in photosystem II (PS_II_). Supplemental UV-B radiation may lead to the light-harvesting pigment protein complex (LH2) of PS_II_ reversible inactivation, and this is responsible for a decreased photosynthetic rate^19^,^65^. With an increase in UV-B, all the Fv/Fm, Y(II), ETR (apparent photosynthetic electron transport rate) and qP (photochemical quenching coefficient) significantly decreased. The results in our study are also consistent with this response. In our study, both the Fv/Fm and Y(II) of *Syntrichia caninervis* followed a pattern of natural shrubs >50% shrubs >0% shrubs. The Fv/Fm and Y(II) of *Syntrichia caninervis* in exposed areas were significantly higher than that of 0% shrubs, despite experiencing the same amount of radiation. There was no significant difference between exposed areas and the natural shrubs in Y(II). This finding may indicate that the shoots of *Syntrichia caninervis* distributed in exposed areas are better adapted to the harsh environment than those present in other habitats^35^,^53^,^66^. The result is different from Haapala *et al*.’s that the cell structure and CO_2_ absorption *of Eriophorum russeolum* were not affected by UV-B, although chlorophyll fluorescence was temporary affected in a three-year experiment^42^.

### The effect of the removal of shrubs on the osmotic adjustment substances

Substances responsible for osmotic adjustments in plants (including vascular plants and cryptogams), including free proline, soluble sugars and soluble protein, are often used as indicators for stress tolerance to drought, temperature and UV-B radiation^67^,^68^,^69^. For example, plants can accumulate soluble sugars and abscisic acid (ABA) to resist acute cold temperatures^17^. In addition, the free proline contents of *Bryum argenteum* and *Didymodon vinealis* in a desertified grassland were significantly higher than those in a typical grassland^32^. Free proline was also found to have accumulated under increased UV-B radiation^20^. In contrast, the soluble protein contents decreased with aggravated stress. Our study is similar to these reports: the soluble sugars and free proline during dry periods (April and August) were significantly higher than those during the snowmelt period. This finding may indicate that *Syntrichia caninervis* is similar to other plants in terms of osmotic adjustment resistance to stress and the accumulated concentrations of soluble sugars and free proline. Naturally, there are also differences between species. Rütten and Santarius^70^ showed that the sucrose content in branches of two mosses (*Plagiomnium affine and Plagiomnium undulatum*) is not related to frost resistance.

As an important regulator of plants, to a certain extent, the soluble protein content reflects plant metabolism, and a higher content reflects a higher plant metabolism. Most previous studies have reported that extreme temperature or drought can decrease activity and reduce metabolism^16^,^71^,^72^. Shi *et al*.^73^ found that soluble protein content decreased with increasing drought stress. Similarly, the soluble protein content of *Syntrichia caninervis* in removed shrub conditions linearly decreased with time. This finding is consistent with the response of chlorophyll fluorescence activity in removed shrub conditions. Furthermore, the activity of *Syntrichia caninervis* was severely diminished with time. However, soluble sugar and free proline exhibited an initial increase and later decrease with a second-order polynomial regression pattern. This finding indicates that *Syntrichia caninervis* can accumulate soluble sugars and free proline to maintain its normal metabolism in the early stages of removal. There are two possible reasons for the changes in osmotic adjustment indices observed in response to the removal of shrubs in the present study. First, plants require more osmotic adjustment substances with an increase in aggravated stress, but it is not sufficient to maintain a source of high concentrations of soluble sugars for the decreased photosynthetic activity. Second, plants typically assign priority to maintaining the survival of the most important plant materials by sacrificing some of the osmotic adjustment substances under limited resource conditions.

Except for the snowmelt periods, the osmotic adjustment substances and antioxidant enzymes of *Syntrichia caninervis* in the 50% shrub removal plots were not significantly different from those in the natural shrub plots. This finding may indicate that partial removal of shade may have a negative impact on the growth of *Syntrichia caninervis*, that snow cover may provide some protection in winter, and that snowmelt in spring may create an opportunity for shrub growth. This response may be an important reason for the subsequent physiological recovery of *Syntrichia caninervis*. Despite the negative influences of partial shrub removal and elimination, there are still significant differences in the plant moisture and chlorophyll fluorescence activity under treatments compared with the natural shrub plots until August.

### The effect of the removal of shrubs on antioxidant enzymes

The cell membrane is the medium of communication between plants and the external environment, and it is also the most important receptor of external environmental signals^74^. The integrity of the cell membrane is directly related to the normal physiological functions of cells. However, most previous studies have reported that plants suffering from stress, such as drought, extreme temperature or UV-B radiation, will produce a large number of reactive oxygen species and upset the balance of cells, causing membrane lipid peroxidation, damaging the cell membrane systems, then threatening the survival of cells^75^,^76^,^77^. With the corresponding stress, plants are usually able to synthesize many antioxidant enzymes, such as SOD, CAT and POD, to remove excessive reactive oxygen species and protect cells from being damaged^78^,^79^,^80^. Many previous studies have reported that the MDA contents and activities of SOD, POD and CAT in plants were significantly increased under water shortage conditions^77^,^81^. The present study also demonstrates that the mosses living in exposed areas exhibited the highest antioxidant enzymes.

Superficially, with the removal of shrubs over time, the environmental stress also increased during the experiment periods. Correspondingly, the antioxidant enzyme activity in *Syntrichia caninervis* was significantly higher under the 0% shrubs treatment than in the other two treatments, whereas there was no significant difference with that in the exposed areas. Does this imply a stronger plasticity of *Syntrichia caninervis*, no matter how environmental changes might influence plant survival? This may not be the case. In this study, the antioxidant enzyme activities were characterized by an initial increase and then a second-order polynomial decrease under the 0% shrub treatments. The most noteworthy result in this study is the significantly higher antioxidant enzyme activity under the 0% shrub treatment compared with the exposed areas during the early stage after treatment. This may indicate that *Syntrichia caninervis* can accumulate antioxidant enzymes in response to the stress in a short-term (4^th^ months). However, this difference gradually disappeared and even exhibited a reverse trend with time (9^th^ months), especially for CAT (Fig. 4a). We acknowledge that the relatively short duration of this study (9^th^ months) might reduce our ability to generalize about how removal shrubs affects the physiological state of the bryophyte in this desert ecosystem. Although the previous studies found that *Syntrichia caninervis* in *exposed areas* are better adapted to the adverse desert environment than to other habitats^48^,^82^, however, there were significant differences in population density, stem height and morphology between microhabitats^83^. It is unclear whether the bryophytes would return to normal levels after removal shrubs in a longer time scale (20–25 years) as did the bog and forest. Because the desert is a very fragile ecosystem compared with other ecosystems^62^. The changes to plant antioxidant enzymes in response to stresses are varied^84^,^85^. Unlike the regression analysis model between the 0% shrub treatment and exposed areas, the 0% shrub treatment and natural or 50% shrub treatment regression analyses are exemplified primarily in two ways. First, the POD and CAT activities are similar to those of osmotic adjustment substances that first increase and then decrease. This response pattern is similar to that of the moss *Hypnum fertile* under a temperature gradient treatment^37^. However, studies have demonstrated that antioxidant enzymes gradually decrease with stress treatments^20^,^86^. In addition, Xie *et al*.^85^ found that crusts increased antioxidant enzymes to protect reactive oxygen species from being damaged. This finding is consistent with the second model in our research, represented by MDA and SOD. There are two possible reasons for the change of antioxidant enzyme activities in response to shrub removal in the present study. First, in response to stress, SOD activity is slightly slower than POD and CAT activity in *Syntrichia caninervis*. Second, SOD may be an important antioxidant enzyme in *Syntrichia caninervis* in response to severe environmental stress, such that the cells sacrifice other materials to ensure the activity of SOD. However, further studies are need to understand the reasons for these responses by antioxidant enzymes to integrated stress.

For the above, the total removal of desert shrubs significantly decreased the chlorophyll fluorescence activity and soluble protein content of *Syntrichia caninervis*, which may threaten their survival. Compared to the osmotic adjustment substances, CAT and POD, SOD may be the most important antioxidant enzymes for *Syntrichia caninervis* in response to severe environmental stress.

## Materials and Methods

### Site description

The study was conducted in the Gurbantunggut Desert (44°11′–46°20′N, 84°31′–90°00′E, 300–600 m altitude), which is located in the center of the Jungger Basin in the Xinjiang Uygur Autonomous Region of China. The Gurbantunggut is the second largest desert in China, covering an area of 4.88 × 10^4^ km^2^. The average annual precipitation in this region is 70–160 mm, the annual evaporation is as high as 2606.6 mm, and the average annual temperature is 7.26 °C, with a greater than 10 °C annual accumulated temperature of approximately 3000–3500 °C. In contrast to other deserts, the Gurbantunggut Desert has a stable snow cover in winter, and the number of snow cover days is approximately 100–150 d, with an average snow depth of approximately 15–20 cm. The dominant plant species include *Haloxylon ammodendron* and *Haloxylon persicum*, with a vegetation cover of less than 30%. Also present are some dwarf shrubs, including *Ephedra distachya, Calligonum eucocladum, Artemisia arenaria*, and *Seriphidium terrae*-*albae*. The snowmelt in spring can promote the germination and growth of annual plants. In addition, three types of well-developed biological crusts, i.e., algae-, lichen-, and moss-dominated crusts, are found in this desert, occupying 28.l7% of the ground surface^87^,^88^.

### Field experimental design and sample collection

**Figure 5:**
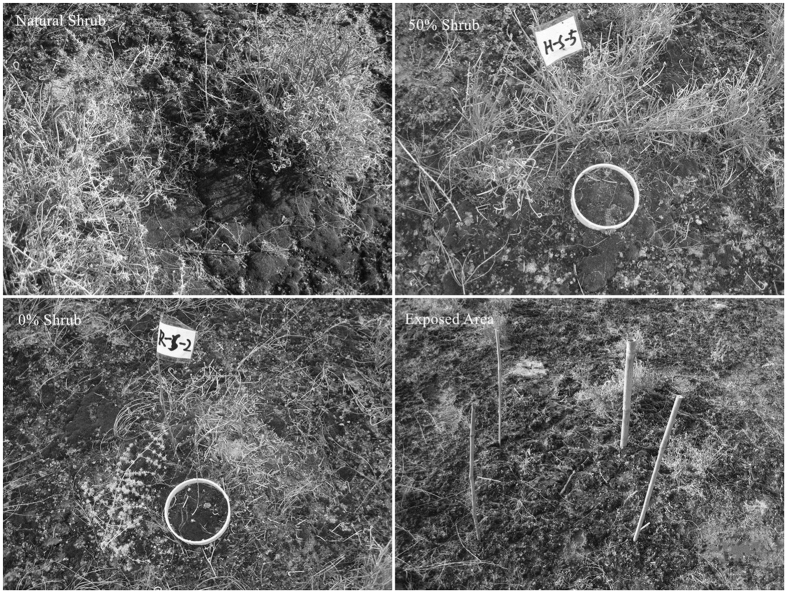
The Schematic diagram of treatments in this study.

The field experiments were conducted near the center of Gurbantunggut Desert (45°24′N, 87°60′E) on November 17, 2013. We selected an interdune area, where the soil surface was covered with well-developed moss crusts (*Syntrichia cainervis* Mitt, according to the herbarium No. 71560 KUN) which lived under the canopy of *Ephedra distachya*, and then established fifteen 2 m × 2 m plots, with three plots each in five blocks. The distance between two plots was greater than 5 m. Three gradients of treatment were applied to the shrubs (total removal of shrubs from the base (0% shrubs, 795.75 ± 13.83 umol·m^−2^·s^−1^), removal of 50% of the shrubs (50% shrubs, 483.58 ± 8.41 umol·m^−2^·s^−1^), natural shrub state (natural shrubs, 198.42 ± 9.80 umol·m^−2^·s^−1^)) in each block. The level of shrub removed were according to the illumination (measured at noon on November 17, 2013 by Li-6400 optical quantum, and five replicates for each treatments). Well-developed moss crusts in exposed areas between shrubs were also identified (Fig. 5). We marked the plots using bamboo sticks.

Ten specimens (each specimen with approximately 100 shoots) of *Syntrichia caninervis* from two treatments (exposed areas and natural shrubs, each treatment included five specimens) were collected on 23 November 2013 (The samples of moss in 0%, 50% shrubs and natural shrubs are the same, due to there were a similar microenvironment before shrubs removed). Twenty specimens from four treatments were collected on 16 January, 17 March, 26 April, and 5 August, 2014. A total of 90 specimens were used for the present experiment. The photosynthetically active leaves and young stems of the sampled moss were rapidly removed using a sharp blade and sieved through a mesh to remove sand and other impurities^23^. The tissues were immediately wrapped with tin foil paper and frozen in liquid nitrogen and then returned to the laboratory and stored at −80 °C prior to analysis^18^.

### Measurements of physiological and biochemical characteristics

#### Measurement of osmotic adjustment substances

We measured the free proline content, soluble sugar content and soluble protein content. The free proline content was determined according to the methods of Bates *et al*.^89^ and Monreal *et al*.^90^ with some modifications. 150 mg of fresh weight of stems and leaves of *Syntrichia caninervis* was weighed on an electronic scale (a precision of 0.001 g), then ground with 5 ml 3% sulfosalicylic acid and extracted for 20 min in boiling water. The extract was transferred to a clean centrifuge tube and clarified by centrifugation at 8,000× g. The supernatant (2 mL) was mixed with 3 ml acetic acid and 3 ml acid ninhydrin. The mixture was oven-incubated 40 min at 100 °C. The reaction mixture was extracted with 5 ml toluene and the extraction allowed to cool. The absorbance values were read at 520 nm, using toluene as a blank, and the standard curve was used to determine the free proline content

The content of soluble sugar was determined using the methods of Lassouane *et al*.^91^. The frozen samples (150 mg) were ground to a fine powder in liquid nitrogen and mixed with 7 mL of 70% ethanol (V/V) for 5 min on ice; the mixture was then centrifuged for 10 min at 4 °C and 8000× g. After adding 1 mL anthrone solution to 200 mL of the extract, the mixture was shaken, heated in a boiling water bath for 10 min and allowed to cool. The absorbance was read at 625 nm, and the soluble sugar content was calculated based on a standard curve.

#### Measurement of antioxidant enzymes

The contents of soluble protein were determined using the methods of Gonzalez and Pignata^38^. Fresh samples (0.150 g) were ground in 5 ml cold (4 °C) deionized water, then clarified by centrifugation at 8,000× g for 30 min at 4 °C; centrifugation was repeated twice. The supernatant was added to a 10 mL volumetric flask. Precisely 200 μL protein extract was drawn from the flask, and 5 mL of Coomassie brilliant blue added, then mixed well. This mix was to stand for 3 min, and the absorbance read at 595 nm.

Malondialdehyde (MDA) and superoxide dismutase (SOD) were determined using the TBA (thiobarbituric acid) and nitroblue tetrazolium methods, respectively^92^,^93^. Peroxidase (POD) and catalase (CAT) were determined using the methods of Fu and Huang^94^.

#### Chlorophyll fluorescence

Chlorophyll fluorescence was measured using a portable modulated fluorometer (PAM-2500, Heinz, Walz, Germany). The saturation pulse method was used to calculate the Fv/Fm. Fo and Fm were measured in the dark after the box in use had been covered for more than 30 min. The parameter settings were based on the recommendations of Zhang *et al*.^58^. The Y(II) of samples were measured at ambient light; saturating pulses were applied to determine the maximal fluorescence yield during actinic illumination, Fm’ and the steady-state level fluorescence during actinic illumination F. The values of Y(II) were calculated by Y(II) = (Fm’−F)/Fm’.

On 17 November, 2013, randomly selected bryophytes under four treatments were used for photosynthetic fluorescence *in situ* measurement. And marked the plots using bamboo sticks. Chlorophyll fluorescence of samples were measured on 23 November, 2013, and on 16 January, 17–25 March, 26 April, and 5 August, 2014. Because of the acute cold temperatures in winter (January) and extremely dry conditions in late spring and summer (April and August), *Syntrichia caninervis* were dormant from dehydration. Thus, during these periods, we did not detect chlorophyll fluorescence activity of *Syntrichia caninervis*. In August 2014, to test the chlorophyll fluorescence, the bryophytes were subjected to a simulated 5 mm rainfall, and the measurement was conducted after one hour of rainfall simulation. In addition, according to previous studies, they were found that the snow-melting period of March was the most important growth period of moss, which accounted for 50% of the total amount of carbon sequestration in the whole year^59^. So in March 2014, the real-time chlorophyll fluorescence was measured at 10:00 am every day using the aforementioned fluorometer *in situ* until no activity was detected.

#### Statistical analyses

Data sets were tested for heteroscedasticity before analyses. Differences in the physiological state between treatments were tested with one-way ANOVA. Temporal differences, overall treatment differences, and their interactions were tested with repeated-measures ANOVA. The LSD test was used for multiple comparisons of treatment effects. All tests were performed using the SPSS19.0 statistical package for Windows (SPSS Inc., Chicago, IL, USA), mapped with Origin 8.0. To model the shape of the influence of the removal of shrubs, the Origin 8.0 software package was used to fit curves between the ratio of change and removal times.

To eliminate the difference between habitats or environments, we placed *Syntrichia caninervis* in exposed areas as an auxiliary control group. We also used the ratio of 0% shrubs to all other groups (50% shrubs, natural shrubs, exposed areas), rather than the absolute values, to characterize the influence of the removal of shrubs on *Syntrichia caninervis* (see formula 1).


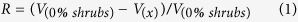


where, *R* represents the rate of change, V represents measured values, and X represents treatments other than removed shrubs (such as 50% shrubs, natural shrubs, exposed areas).

## Additional Information

**How to cite this article:** Yin, B. F. *et al*. Impacts of the removal of shrubs on the physiological and biochemical characteristics of *Syntrichia caninervis* Mitt: in a temperate desert. *Sci. Rep.* **7**, 45268; doi: 10.1038/srep45268 (2017).

**Publisher's note:** Springer Nature remains neutral with regard to jurisdictional claims in published maps and institutional affiliations.

## Figures and Tables

**Table 1:** Repeated measure results on the effects of habitat treatments, periods of measurement, and their interaction on physiological state.

	df	Fluorescence		Osmotic adjustment substance			Antioxidative enzyme			
		Fv/Fm	Y(II)	Soluble sugar	Proline	Soluble protein	MDA	POD	SOD	CAT
F_habitat_	3	4.698*	2.511	7.651**	60.466**	33.742**	109.907**	77.040**	165.744**	46.036**
F_period_	3/4	55.172**	65.841**	997.583**	96.384**	392.859**	685.565**	274.208**	583.458**	480.216**
F_habitat×period_	9/12	7.203**	6.975**	11.180**	14.296**	11.829**	39.305**	5.707**	16.769**	9.673**

Note: Fv/Fm, maximal photochemical efficiency of PS_II_; Y(II), actual PS_II_ efficiency; MDA, malonyldialdehyde; POD, peroxidase; SOD, superoxide dismutase; CAT, catalase. ** and * indicate a significant correlation at 0.01 and 0.05 levels, respectively.

**Table 2:** Regression analysis on the processing time and physiological state of the relative change of three treatments compared with the 0% shrub plots.

Items	0%/Natural^−1^	0%/50%^−1^	0%/Exposed^−1^
Fv/Fm	Y = −0.02X + 0.0196	Y = −0.0082X + 0.0029	Y = −0.0302X + 0.1392
	*R*^2^ = 0.77**	*R*^2^ = 0.4346*	*R*^2^ = 0.6635**
Y(II)	Y = −0.0257X + 0.0296	Y = −0.0152X + 0.0133	Y = −0.0299X + 0.0855
	*R*^*2*^ = 0.7102**	*R*^*2*^ = 0.5468**	*R*^*2*^ = 0.7654**
Soluble protein	Y = −0.0371X − 0.0307	Y = −0.0301X + 0.0018	Y = −0.0993X + 0.5861
	*R*^2^ = 0.6521**	*R*^2^ = 0.6676**	*R*^2^ = 0.6351**
Proline	Y = −0.004X^2^ + 0.026X − 0.019	Y = −0.008X^2^ + 0.062X − 0.032	Y = −0.001X^2^ −0.009X − 0.160
	*R*^2^ = 0.082 ns	*R*^2^ = 0.057 ns	*R*^2^ = 0.474**
Soluble sugar	Y = −0.009X^2^ + 0.072X + 0.027	Y = −0.034X^2^ + 0.275X − 0.065	Y = −0.015X^2^ + 0.040X + 0.568
	*R*^2^ = 0.181 ns	*R*^2^ = 0.319*	*R*^2^ = 0.341*
MDA	Y = 0.019X^2^ − 0.013X + 0.428	Y = −0.001X^2^ + 0.057X + 0.327	Y = −0.004X^2^ + 0.017X + 0.459
	*R*^2^ = 0.330*	*R*^2^ = 0.058 ns	*R*^2^ = 0.030 ns
SOD	Y = 0.005X^2^ + 0.008X − 0.002	Y = 0.002X^2^ + 0.023X − 0.006	Y = −0.007X^2^ + 0.082X − 0.231
	*R*^2^ = 0.839**	*R*^2^ = 0.728**	*R*^2^ = 0.828**
POD	Y = −0.003X^2^ + 0.061X + 0.006	Y = −0.001X^2^ + 0.036X + 0.007	Y = −0.007X^2^ + 0.089X − 0.263
	*R*^2^ = 0.490**	*R*^2^ = 0.480**	*R*^2^ = 0.819**
CAT	Y = −0.012X^2^ + 0.156X + 0.006	Y = −0.010X^2^ + 0.122X + 0.011	Y = −0.009X^2^ + 0.087X − 0.155
	*R*^2^ = 0.619**	*R*^2^ = 0.568**	*R*^2^ = 0.320*

Note: Fv/Fm, maximal photochemical efficiency of PS_II_; Y(II), actual PS_II_ efficiency; MDA, malonyldialdehyde; POD, peroxidase; SOD, superoxide dismutase; CAT, catalase. ** and * indicate a significant correlation at 0.01 and 0.05 levels, respectively.
